# The relationship between neurogenic dysphagia, stroke-associated pneumonia and functional outcome in a cohort of ischemic stroke patients treated with mechanical thrombectomy

**DOI:** 10.1007/s00415-023-11940-7

**Published:** 2023-08-26

**Authors:** Beate Schumann-Werner, Johanna Becker, Omid Nikoubashman, Martin Wiesmann, Jörg B. Schulz, Arno Reich, João Pinho, Cornelius J. Werner

**Affiliations:** 1 Department of Neurology, Medical Faculty, RWTH Aachen University, Pauwelsstrasse 30, 52074 Aachen, Germany; 2 Johanniter Hospital Stendal, Stendal, Germany; 3 Institute of Cognitive Neurology and Dementia Research, Otto Von Guericke University Magdeburg, Magdeburg, Germany; 4 Department of Neuroradiology, Medical Faculty, RWTH Aachen University, Aachen, Germany; 5 JARA-Brain Institute Molecular Neuroscience and Neuroimaging, Research Center Jülich GmbH and RWTH Aachen University, Aachen, Germany

**Keywords:** Stroke, Dysphagia, Endovascular treatment, Thrombectomy, Pneumonia, Outcome

## Abstract

**Introduction:**

Mechanical thrombectomy (MT) is an established treatment approach in acute ischemic stroke patients with large vessel occlusion (LVO). Recent studies suggest that the prevalence of dysphagia and pneumonia risk is increased in this patient population. The aim of this study was to systematically evaluate the prevalence, predictors, and influence of neurogenic dysphagia for 3-month outcome in a large population of patients receiving MT and to elucidate the relationship between dysphagia, stroke-associated pneumonia (SAP) and medium-term functional outcome.

**Materials and methods:**

Data of a prospective collected registry of patients with LVO and MT between 2016 and 2019 were analyzed retrospectively. Binary logistic regression was carried out to determine predictors for dysphagia and 3-month outcome as measured by the modified Rankin Scale, respectively. A mediation analysis was performed to investigate the mediating influence of intercurrent SAP.

**Results:**

A total of 567 patients were included in the study. Mean age was 73.4 years, 47.8% of the patients were female, and median NIHSS was 15.0. The prevalence of dysphagia was 75.1% and 23.3% of all patients developed SAP. In the regression analysis, dysphagia was one of the main independent predictors for poor functional outcome at 3 months. The mediator analysis revealed that the effect of dysphagia on the functional outcome at 3 months was not mediated by the occurrence of SAP.

**Discussion:**

The prevalence of dysphagia is high and exerts both negative short- and medium-term effects on patients with large vessel occlusion who undergo MT.

**Supplementary Information:**

The online version contains supplementary material available at 10.1007/s00415-023-11940-7.

## Introduction

Endovascular treatment or mechanical thrombectomy (MT) is an established treatment approach in acute ischemic stroke (AIS) patients with large vessel occlusion (LVO), resulting in a significantly lower degree of 90-day disability [1, 2]. Dysphagia is a common complication following acute stroke with a reported prevalence of at least 50% depending on diagnostic criteria, evaluation methods and lesion location [3]. Aspiration, the most severe symptom of dysphagia, occurs in approximately 40% of acute stroke patients, the majority of which is silent aspiration due to, for example, reduced sensation or reduced cough [4]. Next to stroke-induced immunosuppression, dysphagia is considered a major cause for stroke-associated pneumonia (SAP), which has been reported in approximately 10% of acute stroke patients [5]. Among post-stroke infections, SAP has the highest impact on relevant outcome parameters, as it increases the risk of mortality, disability and prolongs hospital stays [6, 7]. Functional outcome has been shown to be reduced in association with SAP both on discharge and in long-term follow-up [8, 9]. A recent publication indicates that the risk for SAP is increased after MT, possibly due to endotracheal intubation and prolonged ventilation [10]. Although the relationship between stroke, dysphagia and pneumonia is well established, it is still unknown whether acute ischemic stroke patients receiving MT present a higher risk for aspiration. Also, medium-term effects of dysphagia on functional outcomes are not well studied in this cohort. Finally, the impact of SAP on medium-term functional outcomes has not been examined in patients with LVO undergoing MT. The aim of this study was to systematically evaluate the prevalence, predictors, and influence of neurogenic dysphagia for 3-month outcome in a large population of AIS patients receiving MT and to elucidate the relationship between dysphagia, SAP and medium-term functional outcome.

## Materials and methods

### Data acquisition and patient cohort

We analyzed data of a prospective collected registry of patients with an acute ischemic stroke admitted to a single tertiary center (RWTH Aachen University hospital). We selected adult stroke patients with LVO who had undergone MT between 2016 and 2019 and received a clinical or instrumental dysphagia assessment. The following data were collected from the local registry: demographic information; co-morbidities like hypertension, diabetes mellitus type 2 or atrial fibrillation; clinical and stroke characteristics; stroke severity as depicted by the National Institute of Health Stroke Scale (NIHSS) at admission; size of final infarction in computed tomography (CT) after MT evaluated by the Alberta Stroke Program Early CT Score (ASPECTS) in the last cranial CT before hospital discharge [11]; treatment with intravenous thrombolysis; occurrence of parenchymal hemorrhage type 2 (PH2) as defined by the European Cooperative Acute Stroke Study [12]; successful recanalization (defined as an rTICI score of 2b, 2c or 3); extubation immediately after thrombectomy (yes/no); hours of mechanical ventilation and modified Rankin Scale at 3 months (mRS90). The latter is assessed routinely 3 months after ischemic stroke per phone as part of the information from the local registry. Favorable outcome was defined as mRS 0–2. Additionally to these data, we defined frailty for all patients by calculating the Hospital Frailty Risk Score (HFRS) [13]. For this purpose, the International Classification of Diseases (ICD-10) codes, tenth revision, were retrieved from the hospital information system. The HFRS was specifically developed to identify patients with greater risks of adverse outcomes [13]. It allows categorizing patients as low frailty risk (HFRS < 5), intermediate frailty risk (HFRS 5–15) or high frailty risk (HFRS > 15). Management of AIS patients with LVO in our center follows national and international recommendations and details of the treatment pathways may be found in previous publications from our center [14, 15].

As retrospective chart reviews are prone to coding biases, we performed a comprehensive re-evaluation of the diagnosis *dysphagia* and *pneumonia* for all included patients as follows.

### Dysphagia

After first clinical judgement of the physician, Speech and Language Therapists (SLT) performed a Clinical Bedside Assessment (CBA) including patient status, oral motor assessment and swallow trials with different textures, if possible. Additionally, patients received a Fiberoptic Endoscopic Evaluation of Swallowing (FEES) according to the discretion of the SLT and after discussion with the treating physician. If vigilance was reduced and therefore, a formal swallowing examination could not be performed, the patient was coded as having dysphagia in this study as oral intake was not possible. As per our Standard Operating Procedures, all stroke patients were seen on the day of admission or as soon as possible. Dates of first contact with an SLT were recorded.

### Stroke-associated pneumonia

The diagnosis of pneumonia in this study was based on the modified Centers for Disease Control and Prevention (CDC) criteria for definite SAP [16]: Subjects were required to meet one of the following criteria: fever ≥ 38.3 °C; leukopenia; leukocytosis; for adults > 70 years, a change in mental status for no other reason than pneumonia. Also, two of the following characteristics had to be present: productive cough, dyspnea or tachypnea; purulent sputum or change of characteristics of the sputum; inspiratory crackle or bronchial breathing; abnormal respiratory examination or worsening gas exchange. Pathological X-ray imaging findings were also mandatory. The diagnosis of pneumonia had to occur within 7 days after stroke in order to qualify as SAP. The date of diagnosis was recorded.

### Statistics

In order to visualize the flow and distribution of the critical events, we first created an alluvial diagram for the variables NIHSS, no immediate extubation after MT, dysphagia, SAP and mRS90. For purposes of visualization, we partitioned the NIHSS at admission into quartiles. The alluvial diagram was generated using RawGraphs 2.0 (https://app.rawgraphs.io/). Inferential statistics were performed as follows: SPSS (Version 28; IBM SPSS Statistics) and jamovi 2.2.5 were used for data analysis. Absolute numbers and percentages for nominal and categorical variables were described. To determine significant associations between the dependent variable dysphagia and independent variables, the *χ*^2^ test, Mann–Whitney *U* test or *t*-test were used for univariate analysis. Binary logistic regression was carried out to determine predictors for dysphagia, respectively, 3-month outcome as measured by the modified Rankin Scale (mRS90), binarized into favorable (mRS 0–2) and poor clinical outcomes (mRS > 2). As we were interested in the mediating influence of intercurrent stroke-associated pneumonia (SAP) on functional outcomes, we performed a mediation analysis with dysphagia (among others) being the independent variable (IV), SAP being the mediator and mRS90 serving as the dependent variable. Significance of the respective *a*-pathways from the IVs to the mediator was tested using appropriate univariate statistics. A *p* value < 0.05 was considered statistically significant for all tests.

### Ethics board approval

The study design and protocol based on the local registry of patients with acute ischemic stroke were approved by the local Ethics Committee at RWTH Aachen University, Faculty of Medicine (vote number: EK 335–15). Patient data did not result from additional research activity and were fully anonymized prior to analysis. Thus, no informed consent was necessary according to German legislation and data protection rules.

## Results

### Descriptives

Between 2016 and 2019, a total of 602 AIS patients were admitted in our center and underwent MT. For 35 patients (5.8%), no information regarding dysphagia could be retrieved retrospectively, due to early death or transfer to another hospital before an examination was possible. Therefore, 567 patients were included in the study. Mean age was 73.4 years (SD 13.3), 271 patients were female (47.8%), and median NIHSS was 15.0 (interquartile range [IQR] = 9.0–19.0). Recanalization was successful in 507 (89.4%) of the patients, and 261 patients (46.0%) were treated with intravenous thrombolysis. SAP was observed in 132 patients (23.3%). The incidence of pneumonia was significantly higher in the patient group with versus without dysphagia (29.8% versus 3.5%; *p < *0.001). SAP typically was diagnosed early in the course of the hospital stay (mean day 1.89, SD 1.84).

**Fig. 1:**
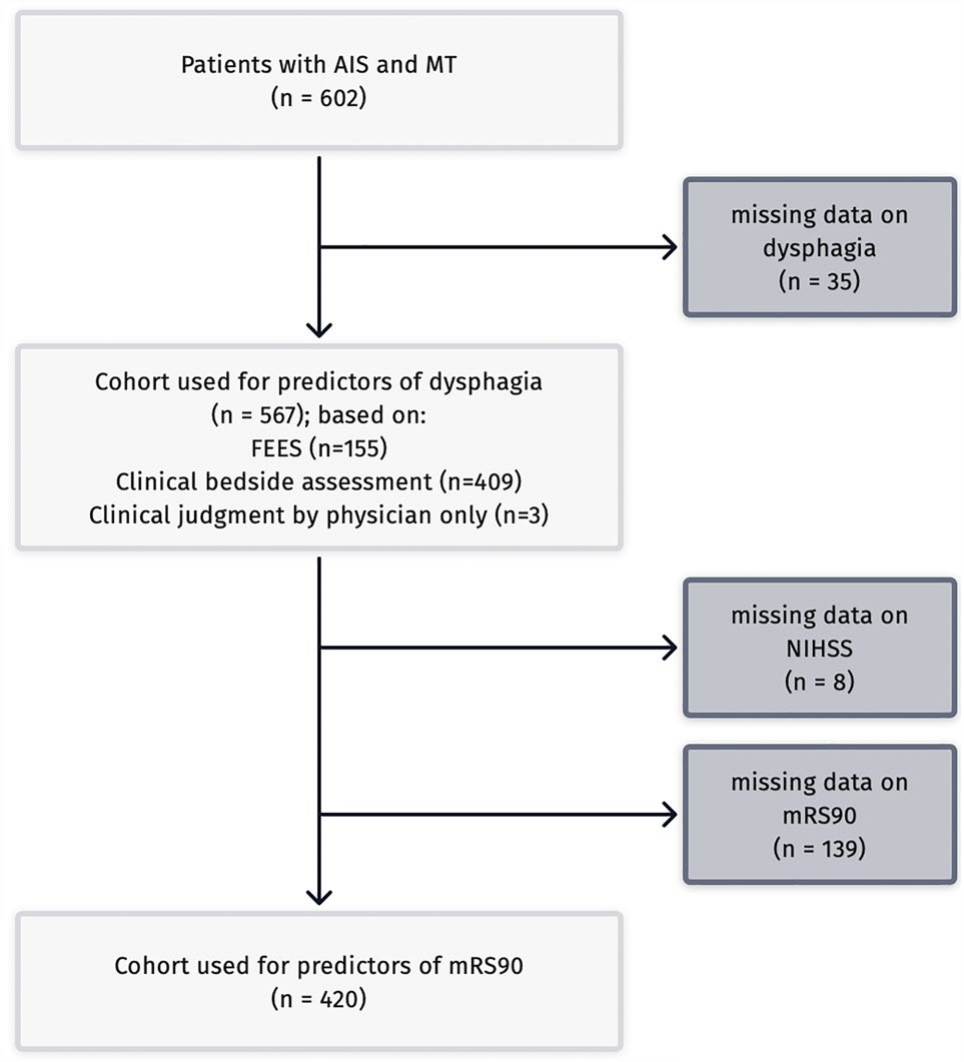
Flow diagram of analyzed patients. *AIS* acute ischemic stroke; *MT* mechanical thrombectomy, *FEES* fiberoptic endoscopic evaluation of swallowing; *CBA* clinical bedside assessment; *mRS90* modified Rankin Scale at 3 months after stroke

In-hospital mortality was 19.8% (*n = *112) and was significantly higher in the group with versus without dysphagia (26.1% versus 0.7%; *p < *0.001). For an overview of the included patients, see Fig. 1.

### Prevalence and predictors for dysphagia

Of the included 567 patients, 426 (75.1%) were diagnosed with dysphagia. Dysphagia diagnosis was either based on FEES by SLT (*n = *155; 27.3% of all cases), CSE by SLT (*n = *409; 72.1%) or clinical judgement by physician only (*n = *3; 0.5%). Time of contact with an SLT was the day of admission or the day after for 92.2% of patients. In the subgroup which received FEES, only 11 patients (7.1%) presented with no dysphagia at all, while 115 patients (74.2%) showed a moderate or severe dysphagia with 40.6% of aspiration according to the Penetration-Aspiration Scale [17]. Patients with dysphagia were significantly older, had a higher risk of frailty according to HFRS, a more severe stroke according to NIHSS, a lower final ASPECTS and a higher prevalence of diabetes mellitus. With respect to recanalization treatment, patients with dysphagia were less frequently extubated immediately after MT and received significantly more ventilation hours. For an overview of the study population and the results of univariate analysis, see Table 1. In the multivariate binary logistic regression, the variables HFRS, age, NIHSS, final ASPECTS and ventilation hours were independent predictors for dysphagia after MT (Table 2).

**Table 1:** Study population and results of univariate analysis for patients with vs. without dysphagia after MT (*n = *567)

Risk factor	Dysphagia *n = *426	No dysphagia *n = *141	*p* value
Sociodemographic characteristics			
Age (years)	74.9 (± 12.8)	68.9 (± 13.71)	** < 0.001**
Male sex	221 (51.9%)	75 (53.2%)	0.787
Frailty			
HFRS	14.4 (10.6–18.8)	8.7 (6.2–12.3)	** < 0.001**
Stroke severity and characteristics			
NIHSS at admission	16.0 (12.0–20.0)	8.0 (4.0–15.0)	** < 0.001**
Final ASPECTS	6.0 (4.0–8.0)	8.0 (7.0–9.0)	** < 0.001**
Parenchymal hemorrhage type 2	18 (4.2%)	2 (1.4%)	0.117
Vertebrobasilar territory	52 (12.2%)	13 (9.2%)	0.335
Cardioembolism	238 (55.9%)	79 (56.0%)	0.974
Comorbidities			
Hypertension	352 (82.6%)	107 (75.9%)	0.077
Atrial fibrillation	206 (48.4%)	65 (46.1%)	0.625
Diabetes mellitus	125 (29.3%)	29 (20.6%)	** < 0.001**
Dyslipidemia	116 (27.2%)	45 (31.9%)	0.285
Previous stroke	82 (19.3%)	19 (13.5%)	0.118
Recanalization treatment			
Successful recanalization	376 (88.3%)	131 (92.9%)	0.120
Intravenous thrombolysis	187 (43.9%)	74 (52.5%)	0.076
No extubation after MT	304 (71.4%)	64 (45.4%)	** < 0.001**
Ventilation duration (hours)	155.2 (± 292.0)	6.27 (± 49.6)	** < 0.001**

*p*-values less than the predefined threshold of < 0.05 are highlighted in bold

Values are shown as *n* (%), median (P25-P75) or mean (± SD). Missing data for: NIHSS (*n = *8), ASPECTS (*n = *3), previous stroke (*n = *1); Atrial fibrillation (*n = *1); level of significance *p < *0.05 based on Chi-Square test for nominal variables, Mann–Whitney *U* test for ordinal variables, *t*-test for continuous variables

*MT* mechanical thrombectomy, *HFRS* Hospital Frailty Risk Score, *NIHSS* National Institutes of Health Stroke Scale, *ASPECTS* Alberta Stroke Program Early CT

**Table 2:** Multivariate binary logistic regression of possible predictors for dysphagia after MT (*n = *567)

Predictor	OR (95% CI)	*p* value
HFRS (per 1-point increase)	1.16 (1.10–1.22)	** < 0.001**
Age (per 1-year increase)	1.03 (1.01–1.05)	**0.001**
Diabetes mellitus	1.93 (1.00–3.73)	0.051
NIHSS at admission (per 1-point increase)	1.10 (1.06–1.15)	** < 0.001**
Final ASPECTS (per 1-point increase)	0.66 (0.57–0.77)	** < 0.001**
Ventilation duration (per 1-h increase)	1.01 (1.00–1.02)	**0.028**
No extubation after MT	1.49 (0.88–2.53)	0.142

*p*-values less than the predefined threshold of < 0.05 are highlighted in bold

Missing data for: NIHSS (*n = *8), ASPECTS (*n = *3); level of significance *p < *0.05

*MT* mechanical thrombectomy, *HFRS* Hospital Frailty Risk Score, *NIHSS* National Institutes of Health Stroke Scale, *ASPECTS* Alberta Stroke Program Early CT, *OR* odds ratio, *CI* confidence interval

### Chronological flow of the critical events

**Fig. 2:**
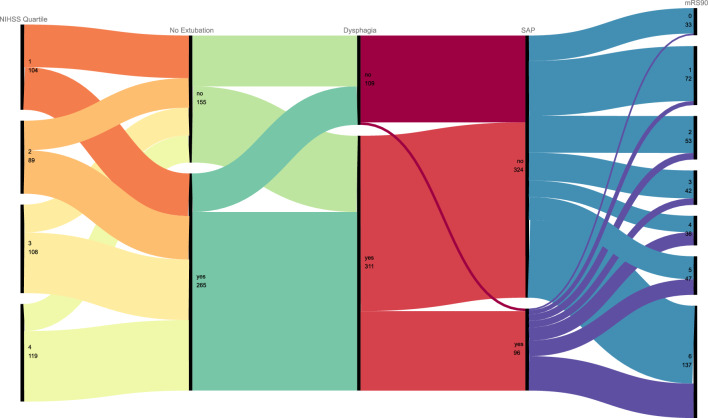
Alluvial diagram of the critical clinical events and outcomes (*n = *420); NIHSS Quartile: National Institutes of Health Stroke Scale Quartile, quartile ranges: *Q1*: < 9, *Q2*: 9–14; *Q3*: 15–18, *Q4*: > 19; *SAP* stroke-associated pneumonia; *mRS90* modified Rankin Scale at 3 months after stroke

It is apparent from the alluvial diagram (Fig. 2) that a substantial proportion of patients was not extubated immediately after MT. The proportion slightly increases with increasing NIHSS scores. Still, no NIHSS group is exempt from failure to extubate. A significant proportion of both groups goes on to be diagnosed with dysphagia, with a higher percentage in the “no extubation” stream. Crucially, almost no patient without dysphagia goes on to develop SAP, while this is the case for a substantial part of the patients with dysphagia. From the graphic, it seems as if a large proportion of patients with SAP progresses to a poor medium-term functional outcome, while this is more evenly distributed in the “no SAP” cohort. From this graphic, it becomes clear that both dysphagia as well as SAP are important crossroads in the patient trajectory. However, this does not necessarily imply causality or significant association. These were tested in the following analyses.

### Functional outcome at 3 months after stroke

Modified Ranking Scale scores at 3 months after stroke were collected per phone as part of the information from the local registry. From the original cohort, mRS90 could be retrieved for 420 patients with complete NIHSS. A favorable outcome occurred in 160 patients (37.5%), while a poor outcome was found in 267 patients (62.5%). A comparison of the two groups showed many known factors and comorbidities to be associated with poor 3-month outcome (see Supplementary material Table S1). Importantly, patients with a poor 3-month outcome had significantly higher rates of dysphagia and SAP. In the multivariate binary logistic regression, the variables age, HFRS, final ASPECTS, dysphagia, no extubation after MT, ventilation hours and no successful recanalization were independent predictors for a poor 3-month outcome (Table 3). The mediator analysis revealed that the effect of dysphagia on the functional outcome was not mediated by the occurrence of stroke-associated pneumonia (see Supplementary material Table S2).

**Table 3:** Multivariate binary logistic regression of possible predictors for poor functional outcome at 3 months (*n = *420)

Predictor	OR (95% CI)	*p* value
NIHSS at admission (per 1-point increase)	1.02 (0.98–1.07)	0.286
Age (per 1-year increase)	1.06 (1.04–1.09)	** < 0.001**
Hypertension	1.76 (0.82–3.77)	0.149
Diabetes mellitus	0.92 (0.47–1.80)	0.809
HFRS (per 1-point increase)	1.05 (1.00–1.11)	**0.039**
Final ASPECTS (per 1-point increase)	0.72 (0.61–0.83)	** < 0.001**
SAP	1.00 (0.47–2.13)	0.992
Dysphagia	3.51 (1.69–7.28)	** < 0.001**
Ventilation duration (per 1-h increase)	1.00 (1.00–1.01)	**0.012**
No extubation after MT	2.43 (1.35–4.36)	**0.003**
Atrial fibrillation	1.18 (0.63–2.19)	0.604
Intravenous thrombolysis	0.81 (0.46–1.42)	0.460
Successful recanalization	0.30 (0.09–0.94)	**0.038**
Parenchymal hemorrhage type 2	3.31 (0.34–31.90)	0.301
Vertebrobasilar territory	2.50 (0.95–6.55)	0.063
Previous stroke	1.85 (0.85–4.04)	0.124

*p*-values less than the predefined threshold of < 0.05 are highlighted in bold

Level of significance *p < *0.05

*NIHSS* National Institutes of Health Stroke Scale, *HFRS* Hospital Frailty Risk Score, *ASPECTS* Alberta Stroke Program Early CT; *SAP* stroke-associated pneumonia; *MT* mechanical thrombectomy, *OR* odds ratio; *CI* confidence interval

## Discussion

To our knowledge, this is the first study examining the complex relationship between factors predicting dysphagia after endovascular stroke treatment, its role in stroke-associated pneumonia and in predicting functional outcome at 3 months in a large patient cohort. Even though the majority of patients received their diagnosis based on a clinical assessment, we provide a substantial number of instrumental diagnostics, supporting the high prevalence in this patient population. In our cohort, 155 patients (27.3%) received FEES. In this selected subgroup, 115 patients (74.2%) showed a moderate or severe dysphagia. This high rate of dysphagia has already been described previously in a much smaller cohort [18]. These rates exceed the reported prevalence of dysphagia after stroke in a general population, even when using instrumental testing like FEES [3, 19]. It has to be considered that only indirect comparisons can be made as long as no study exists comprising both MT and no-MT cohorts. This of course is difficult for a variety of reasons, both ethical and methodological.

The high prevalence for dysphagia after MT is probably multifactorial: First of all, the brain areas that are most susceptible to damage by a large vessel occlusion such as insula and internal capsule have been shown to be essentially involved in swallowing function [20]. Secondly, the treatment itself can contribute to the prevalence of dysphagia: as is standard in many centers, all patients in our sample underwent MT under general anesthesia. As for now, it is still unclear whether this approach leads to poorer functional outcome at 3 months in comparison with conscious sedation [21]. In a previous study, shorter ventilation time was associated with lower pneumonia rate and better clinical outcome [22]. Accordingly, the duration of ventilation is a predictor for dysphagia in our cohort, in addition to patient-related factors such as age, frailty and clinical and imaging markers of stroke severity.

More importantly, the presence of dysphagia itself is a predictor of further negative outcomes. Notably, it is an independent predictor of a poor functional outcome at three months. This relationship has been previously demonstrated in other stroke populations using both clinical as well as instrumental diagnostics [20, 23–25]. As such, we demonstrate that this relationship also holds for patients undergoing MT. Considering the high rate of dysphagia in our cohort, this has important implications for prognosis and management after discharge. Our data do not allow any conclusions with respect to the mechanism, however. This should be studied prospectively. One potential factor could be the occurrence of SAP, which we will discuss in the following paragraph.

SAP is a known complication of stroke and dysphagia. Previously, the time between stroke without MT and the occurrence of SAP has been described to be in the range of 3–4 days [26]. It is noteworthy that in our cohort, SAP occurred earlier (day 1.89 on average). There is still the caveat that no direct comparisons can be made between these cohorts. However, one could speculate that the severity of the stroke and components of the therapy (such as intubation) contribute to this shifted temporal profile. Still, SAP occurred after diagnosis of dysphagia in our sample, suggesting a causal mechanism. Using mediator analysis techniques, we show that, although dysphagia is strongly related to SAP in our cohort, the detrimental effect of dysphagia on the functional outcome after 3 months is not mediated by the occurrence of SAP. This means that neurogenic dysphagia after stroke exerts both negative short- and medium-term effects on patients with LVO undergoing MT, but both occur independently of one another. Therefore, our data suggest that identifying dysphagia and successfully preventing SAP will not be enough to improve outcomes after stroke, as other factors, such as dysphagia-associated functional status or nutritional aspects, may play an additional role.

One interesting finding is the role of frailty as predictor for medium-term functional outcome in relation to dysphagia. Frailty is defined by a cumulative physiological decline and loss of resistance when dealing with stressors [27]. In a previous study, we showed that high frailty risk measured by a HFRS > 15 points was associated with poor 3-month outcome in stroke patients who received endovascular treatment [28]. In our multivariate logistic regression model, frailty is still an independent predictor even in the presence of dysphagia. Several studies have reported strong associations between dysphagia and frailty, suggesting that dysphagia is a main risk factor for frailty due to, e.g., nutritional status [29]. In our cohort, both are independent predictors for 3 month functional outcome. Therefore, the interrelation between frailty and dysphagia in stroke and LVO is still unclear and needs further investigation.

Due to the retrospective design of our study, potential confounding variables could not be fully controlled. One particularly challenging aspect of the study is the substantial proportion of patients classified as having dysphagia due to their reduced state of consciousness on the basis of the argument that they are not investigable. However, two arguments speak against this view: First, vigilance and dysphagia are closely interrelated. Obviously, vigilance is a necessary precondition for successful swallowing particularly with respect to the oral phase of swallowing. Several studies have shown that oral feeding is related to the level of consciousness in individuals with brain injuries [30, 31]. On top of that, however, one recent cohort study examined the incidence of dysphagia in individuals with disorders of consciousness using objective measurements like FEES and importantly reported that 99% of the subjects showed swallowing abnormalities also in the pharyngeal phase of swallowing [32]. Given this finding, it seems reasonable to assume that patients with reduced consciousness need to be classified as dysphagic by necessity. The second argument pertains to the frequency and clinical importance of considering these patients. Within the group that received clinical bedside assessment, 105 patients (25.7%) had reduced consciousness. This illustrates that these patients are clearly part of daily routine, and their outcomes need to be assessed, too. Excluding them would shift our sample substantially toward the less severely affected cases. In any case, leaving them out from our analyses did not change the results in a major way (sensitivity analysis, data not shown).

One potentially important limitation considering global generalization of our findings stems from the fact that all our patients underwent MT with general anesthesia and intubation. In our data, this has a clear impact on dysphagia and medium-term functional outcome. Our study cannot infer on settings where conscious sedation is employed. It would be highly interesting to compare both methods with each other regarding the prevalence of dysphagia and medium-term functional outcomes.

Also, no information on the duration or resolution of dysphagia was available, as were data on premorbid mRS values as well as body weight before and after stroke. It can be speculated that these factors could have an additional influence on our outcome parameters of interest. This should be addressed in prospective studies.

With respect to attrition bias, it has to be noted that 139 patients were lost to follow-up (24.5%), which is substantial. Additional analyses revealed that these patients were slightly more frail (HFRS 15.0 vs. 13.6; *p = *0.019) but had a slightly better mRS on discharge (3.3 vs. 3.8, *p = *0.022). Additionally, age, ASPECT scores, NIHSS or prevalence of dysphagia did not differ significantly (data not shown). Still, this limitation has to be acknowledged.

It is interesting to note that NIHSS on admission is not a predictor of functional outcome at three months. Considering that successful recanalization and final infarction size are independent predictors of functional outcome, this is a highly plausible finding and underlines the benefit of MT in LVO patients, even those presenting with clinically severe stroke.

In order to secure the maximal benefit of MT, it seems plausible that efforts to efficiently and comprehensively manage dysphagia should be pursued also in the long run, given that dysphagia is highly prevalent and seems to play an important role in determining medium-term outcomes after MT.

## Conclusion

Our study shows that the incidence of dysphagia is very high in patients who received endovascular stroke treatment, particularly when (indirectly) compared to a general stroke population. The role of dysphagia is emphasized even more given the fact that it has both negative short-term effects (SAP) and a significant impact on medium-term functional outcome, independently from SAP. Structured dysphagia management programs both in and after discharge from hospital should be evaluated regarding their effect on functional outcomes after LVO and MT.

### Supplementary Information

Below is the link to the electronic supplementary material.

Supplementary file1 (DOCX 27 KB)

## Data Availability

Data not provided in the article may be shared (anonymized) at the request of any qualified investigator for purposes of replicating procedures and results.
