# Supplementary material for: The relationship between neurogenic dysphagia, stroke-associated pneumonia and functional outcome in a cohort of ischemic stroke patients treated with mechanical thrombectomy
Source: J Neurol. 2023 Aug 26;270(12):5958–65. doi: 10.1007/s00415-023-11940-7 (PMC10632272; doi:10.1007/s00415-023-11940-7)
Supplement: Supplementary file 1 — Supplementary file1 (DOCX 27 KB) [file 415_2023_11940_MOESM1_ESM.docx]

**Supplementary Material**

**Table S1: Results of univariate analysis for patients with favourable (mRS 0-2) vs. poor (mRS >2) functional outcome at 3 months (n=420)**

| **Risk factor** | **mRS 0-2**  **n = 158** | **mRS >2**  **n = 262** | **p-value** |
| --- | --- | --- | --- |
| **Sociodemographic characteristics** |  |  |  |
| Age | 68.5 (±12.8) | 76.5 (± 12.9) | **<0.001** |
| Male sex | 93 (58.9%) | 133 (50.8%) | 0.107 |
| **Frailty** |  |  |  |
| HFRS | 9.0 (6.3-14.0) | 14.4 (10.9-18.3) | **<0.001** |
| **Stroke severity and characteristics** |  |  |  |
| NIHSS at admission | 10.0 (6.0-16.0) | 17.0 (12.0-20.0) | **<0.001** |
| Final ASPECTS | 8.0 (6.0-9.0) | 6.0 (3.0-8.0) | **<0.001** |
| Parenchymal hemorrhage type 2 | 1 (0.6%) | 15 (5.7%) | **0.008** |
| Vertebrobasilar territory | 12 (7.6%) | 38 (14.5%) | **0.034** |
| Cardioembolism | 81 (51.3%) | 150 (57.3%) | 0.232 |
| **Comorbidities** |  |  |  |
| Hypertension | 117 (74.1%) | 220 (84.0%) | **0.013** |
| Atrial fibrillation | 63 (39.9%) | 138 (52.9%) | **0.010** |
| Diabetes mellitus | 29 (18.4%) | 78 (29.8%) | **0.009** |
| Dyslipidemia | 50 (31.6%) | 69 (26.3%) | 0.242 |
| Previous stroke | 20 (12.7%) | 54 (20.7%) | **0.037** |
| **Recanalization treatment** |  |  |  |
| Successful recanalization | 151 (95.6%) | 227 (86.6%) | **0.003** |
| Intravenous thrombolysis | 88 (55.7%) | 116 (44.3%) | **0.023** |
| No extubation after MT | 68 (43.0%) | 197 (75.2%) | **<0.001** |
| Ventilation hours | 19.1 (± 132.1) | 168.9 (± 283.7) | **<0.001** |
| **Complications**  Dysphagia  SAP | 72 (45.6%)  15 (9.5%) | 239 (91.2%)  81 (30.9%) | **<0.001**  **<0.001** |

Values are shown as n (%). median (P25-P75) or mean (± SD); MT: Mechanical thrombectomy. HFRS: Hospital Frailty Risk Score. NIHSS: National Institutes of Health Stroke Scale. ASPECTS: Alberta Stroke Program Early CT; SAP: stroke-associated pneumonia; Missing data for: ASPECTS (n=3). previous stroke (n=1); Atrial fibrillation (n=1); level of significance p <0.05 based on Chi-Square test for nominal variables. Mann-Whitney-U test for ordinal variables. t-Test for continuous variables

**Table S2: Mediator analysis of dysphagia. stroke-associated pneumonia and functional outcome at 3 months (n=420)**

|  | | | | 95% C.I. (a) | |  | | | |
| --- | --- | --- | --- | --- | --- | --- | --- | --- | --- |
| Type | **Effect** | **Estimate** | **SE** | **Lower** | **Upper** | **β** | **z** | **p** |  |
| Indirect | Extubation ⇒ SAP ⇒ mRS90 | -0.00893 | 0.01933 | -0.04682 | 0.02895 | -0.002 | -0.462 | 0.644 |  |
|  | PH2 ⇒ SAP ⇒ mRS90 | 0.0088 | 0.02111 | -0.03257 | 0.05018 | 0.000783 | 0.417 | 0.677 |  |
|  | NIHSS ⇒ SAP ⇒ mRS90 | -3.20e−4 | 0.000727 | -0.00175 | 0.00111 | -0.00114 | -0.439 | 0.66 |  |
|  | ASPECTS ⇒ SAP ⇒ mRS90 | -1.56e−4 | 0.000856 | -0.00183 | 0.00152 | -1.90e−4 | -0.183 | 0.855 |  |
|  | Ventilation hours ⇒ SAP ⇒ mRS90 | 0.0000486 | 0.000103 | -1.54e−4 | 0.000251 | 0.00562 | 0.471 | 0.637 |  |
|  | HFRS ⇒ SAP ⇒ mRS90 | 0.00124 | 0.00264 | -0.00394 | 0.00641 | 0.00362 | 0.469 | 0.639 |  |
|  | Dysphagia ⇒ SAP ⇒ mRS90 | 0.01214 | 0.02621 | -0.03924 | 0.06351 | 0.00246 | 0.463 | 0.643 |  |
| Direct | **Extubation ⇒ mRS90** | -0.76844 | 0.17612 | -1.11362 | -0.42326 | -0.17183 | -4.363 | **< .001** |  |
|  | **PH2 ⇒ mRS90** | 0.8883 | 0.43033 | 0.04487 | 1.73174 | 0.07906 | 2.064 | **0.039** |  |
|  | **NIHSS ⇒ mRS90** | 0.02309 | 0.01163 | 0.000294 | 0.04589 | 0.08204 | 1.985 | **0.047** |  |
|  | **ASPECTS ⇒ mRS90** | -0.16449 | 0.03412 | -0.23137 | -0.09761 | -0.19939 | -4.821 | **< .001** |  |
|  | Ventilation hours ⇒ mRS90 | 0.00053 | 0.000369 | -1.94e−4 | 0.00125 | 0.06126 | 1.436 | 0.151 |  |
|  | HFRS ⇒ mRS90 | 0.02578 | 0.01478 | -0.00318 | 0.05474 | 0.07538 | 1.745 | 0.081 |  |
|  | **Dysphagia ⇒ mRS90** | 1.72809 | 0.22968 | 1.27793 | 2.17826 | 0.35076 | 7.524 | **< .001** |  |
| Total | **Extubation ⇒ mRS90** | -0.77738 | 0.17536 | -1.12107 | -0.43369 | -0.17383 | -4.433 | **< .001** |  |
|  | **PH2 ⇒ mRS90** | 0.8971 | 0.43056 | 0.05322 | 1.74099 | 0.07984 | 2.084 | **0.037** |  |
|  | **NIHSS ⇒ mRS90** | 0.02277 | 0.01163 | -2.02e−5 | 0.04557 | 0.0809 | 1.958 | **0.05** |  |
|  | **ASPECTS ⇒ mRS90** | -0.16465 | 0.03417 | -0.23162 | -0.09768 | -0.19958 | -4.818 | **< .001** |  |
|  | Ventilation hours ⇒ mRS90 | 0.000579 | 0.000355 | -1.17e−4 | 0.00128 | 0.06688 | 1.63 | 0.103 |  |
|  | HFRS ⇒ mRS90 | 0.02702 | 0.01456 | -0.00153 | 0.05557 | 0.079 | 1.855 | 0.064 |  |
|  | **Dysphagia ⇒ mRS90** | 1.74023 | 0.22857 | 1.29224 | 2.18823 | 0.35322 | 7.613 | **< .001** |  |

ASPECTS: Alberta Stroke Program Early CT. HFRS: Hospital Frailty Risk Score. mRS90: modified Rankin Scale at 90 days after stroke. NIHSS: National Institutes of Health Stroke Scale. PH2: Parenchymal hemorrhage type 2; SAP: stroke-associated pneumonia; SE: standard error; C.I.: confidence interval; level of significance p <0.05
